# Gene hydrogel platforms for targeted skin therapy: bridging hereditary disorders, chronic wounds, and immune related skin diseases

**DOI:** 10.3389/fddev.2025.1598145

**Published:** 2025-07-01

**Authors:** Liangtao Li

**Affiliations:** School of Medicine, Jinan University, Guangzhou, China

**Keywords:** gene hydrogel, gene delivery, hereditary skin disease, wound healing, immune related skin disease

## Abstract

Gene therapy, a pivotal cornerstone in biomedical research, has emerged as a transformative approach for addressing a wide spectrum of dermatologic conditions, including hereditary disorders, chronic wounds, and immune related skin diseases. The skin, with its expansive surface area and regenerative capacity, serves as an ideal platform for localized gene delivery. However, conventional gene therapy strategies face critical limitations, such as high costs, suboptimal transfection efficiency, immunogenicity, and off-target effects. In this context, gene hydrogels have emerged as an innovative paradigm, offering tailored physicochemical and biological functionalities to overcome these challenges. Gene hydrogels are distinguished by their tunable morphologies (e.g., particulate or bulk gel configurations), which enable precise control over therapeutic release kinetics and spatial distribution. Their three-dimensional polymeric networks recapitulate the extracellular matrix, functioning as bioactive scaffolds that enhance tissue regeneration, facilitate cell migration, and accelerate wound healing. By integrating stimuli-responsive polymers, these hydrogels achieve spatiotemporal control of gene delivery, improving target specificity while minimizing systemic exposure. Furthermore, their inherent biocompatibility and biodegradability mitigate immunogenic risks and prevent long-term residue accumulation, addressing pivotal safety concerns in clinical translation. This review systematically examines the multifaceted advantages of gene hydrogels, including their ability to bypass the stratum corneum barrier, protect genetic payloads from enzymatic degradation, and sustain localized therapeutic effects over extended periods. Recent advancements in “smart” hydrogels, responsive to pathological cues such as pH fluctuations or matrix metalloproteinase overexpression, further underscore their potential in personalized medicine. By synergizing material science with gene-editing technologies, gene hydrogels represent a revolutionary leap toward precision dermatologic therapies. Future challenges, such as scalable manufacturing and dynamic regulatory mechanisms, are critically analyzed alongside opportunities in intelligent material design and interdisciplinary innovation. This comprehensive analysis positions gene hydrogels as a cornerstone for next-generation dermatologic therapeutics, bridging the gap between laboratory innovation and clinical impact.

## 1 Introduction

### 1.1 Gene delivery as a promising therapeutic approach

The skin is a dynamic defense system with a complex structure, primarily composed of keratinocytes in the epidermis that form the outermost protective layer through tight junctions and corneodesmosomes (Søgaar et al., 2021). This intricate architecture poses challenges to the penetration of macromolecular therapeutics (Subramanian et al., 2024), including nucleic acid-based agents such as plasmid DNA, mRNA, and CRISPR ribonucleoproteins (Zhang W. et al., 2024). The stratum corneum, characterized by its hydrophilic nature and anionic surface charge, further impedes passive diffusion of cationic delivery carriers, such as polyethyleneimine (PEI) and liposomes (Singh et al., 2022). Additionally, cutaneous nucleases and proteases rapidly degrade unprotected genetic payloads, with naked mRNA exhibiting a half-life of less than 30 min on the skin surface (Chen et al., 2022).

Current gene delivery systems, including viral and non-viral vectors, have demonstrated notable mechanistic advantages but are also confronted with significant technological limitations. Viral vectors, particularly γ-retroviral vectors (RVs) and self-inactivating lentiviral (SIN-LV) platforms, have demonstrated notable clinical efficacy. Pioneering work by Siprashvili et al. (2016) established proof-of-concept through successful epidermal regeneration using RV-transduced autologous keratinocyte grafts in junctional epidermolysis bullosa patients. Subsequent clinical translation was evidenced in two registered trials (NCT02493816, NCT02810951) employing SIN-LV-mediated *COL7A1* transduction in patient-derived fibroblasts, which achieved durable type VII collagen restoration via intradermal transplantation. (Lwin et al., 2019). Recent preclinical advances by Donadon et al. (2019) demonstrated the therapeutic potential of adeno-associated virus serotype 9 (AAV9) vectors through SPINK5 gene delivery in a Netherton syndrome murine model, resulting in functional recovery of epidermal barrier integrity. Nevertheless, critical challenges persist across viral vector platforms, including host immune responses, potential insertional oncogenesis, limited transgene cargo capacity, and inefficient *in vivo* delivery kinetics - factors that collectively impede broad clinical implementation (Bae and Park, 2020; Rubin, 2020; Wang et al., 2022; Woodworth, 2020).

Concurrently, non-viral vector systems have garnered significant scientific interest as versatile alternatives for cutaneous gene therapy applications. Polyethylenimine (PEI)-based architectures maintain their status as gold-standard polymeric vectors, with extensive preclinical characterization of their nucleic acid complexation dynamics and endosomal escape mechanisms (Søgaar et al., 2021). Recent technological innovations have propelled lipid-based platforms to clinical relevance: Eden et al. (Subramanian et al., 2024) demonstrated that locally administered LNP-encapsulated mRNA induced tumor-specific T-cell responses in a Phase I clinical trial (NCT04882718). Complementing this, Hsu et al. (Zhang W. et al., 2024) developed ionizable lipid nanoparticles capable of sustained *COL7A1* mRNA delivery, achieving durable type VII collagen restoration (4 weeks) in patient-derived keratinocyte cultures. Comparative analyses reveal that non-viral systems demonstrate distinct advantages over viral counterparts, including enhanced biocompatibility, cost-effective manufacturing workflows, expanded genetic cargo capacity (>20 kb), and precise dose modulation capabilities (Singh et al., 2022; Sun et al., 2023). However, persistent translational barriers persist, particularly heterogeneous transfection efficiency and inadequate stratum corneum penetration kinetics, underscoring the need for rigorous clinical optimization to achieve therapeutic equivalence with viral platforms (Chen et al., 2022).

Currently, the development of gene therapy products has encountered significant challenges, primarily due to the lack of safe and efficient delivery systems and the urgent need for minimally invasive administration routes (Søgaar et al., 2021). In most laboratory and preclinical studies, genetic materials are typically dissolved in alkaline buffer solutions and directly applied to the skin without any formulation additives (Subramanian et al., 2024; Zhang W. et al., 2024; Singh et al., 2022; Sun et al., 2023; Blair et al., 2020). However, this conventional approach presents several critical limitations: (i) inadequate control over drug pharmacokinetics, (ii) occurrence of acute toxicity, (iii) poor tissue retention of therapeutic agents, (iv) suboptimal therapeutic outcomes, and (v) inconsistent translation from preclinical to clinical results. These limitations underscore the critical necessity of developing advanced drug delivery systems tailored for gene therapy. To address these challenges, a hydrogel-based gene delivery platform has emerged as a novel therapeutic strategy in dermatology. By synergistically integrating advancements in material science and gene-editing technologies, this innovative platform is poised to establish a transformative roadmap for next-generation personalized dermatological treatments. In the following sections, we systematically analyze the unique advantages of hydrogels as gene carriers, including their tunable physicochemical properties, sustained release kinetics, and enhanced biocompatibility. Furthermore, we critically evaluate their therapeutic potential in overcoming current limitations of cutaneous gene delivery, such as stratum corneum penetration barriers and nuclease-mediated payload degradation. These insights provide a foundation for developing innovative solutions to advance precision medicine in dermatology.

### 1.2 Gene hydrogel: an innovative challenge in gene delivery

In recent years, gene delivery systems for dermatological applications have advanced rapidly, with hydrogels emerging as a promising solution to the challenges posed by the skin barrier. Hydrogels are three-dimensional, cross-linked polymeric networks that provide a unique platform for encapsulating genetic payloads (Sun et al., 2023). They enable sustained release through controlled diffusion, matrix expansion, or degradation kinetics, thereby circumventing the rapid clearance of free carriers and offering enhanced protection against enzymatic degradation and oxidative stress (Zhao et al., 2022).

Hydrogels address key challenges in gene delivery through dual functionality. Microneedle-incorporated formulations mechanically breach the stratum corneum to establish intradermal drug depots, while viscoelastic hydrogel dressings enhance follicular and transappendageal delivery via conformal skin adhesion (Zeng et al., 2021). Additionally, hydrogels fabricated from biocompatible materials such as hyaluronic acid and collagen possess intrinsic anti-inflammatory properties, which can reduce immune recognition and minimize adverse immune responses (Lu et al., 2023). As shown in Table 1, these attributes position hydrogels as a superior alternative to traditional free carriers (Bischof and Hierl, 2024).

**TABLE 1:** Challenges of free carriers and hydrogels in the field of gene delivery.

Challenge	Free vectors	Gene hydrogels
Stratum corneum penetration	Passive diffusion, insufficient efficiency	Microneedle-assisted + adhesion penetration, high efficiency
Enzymatic degradation	Nucleic acids are susceptible to rapid degradation by DNase/RNase	Network isolation protection with extended half-life
Immune activation	High risk of TLR/complement pathway activation	Physical barrier + anti-inflammatory material, reduced inflammatory factors
Targeting	Systemic exposure, significant off-target effects	Local sustained release + environmental response, Excellent target cell delivery efficiency
Repeat dosing requirements	Frequent injections (e.g., once a day)	A single application lasts for more than 7 days

Through continuous optimization, hydrogel-mediated gene delivery has transformative potential for advancing precision medicine in dermatology. By overcoming the limitations of free carriers, such as short retention time, off-target effects, and immune activation, hydrogels are expected to expand beyond the treatment of rare skin diseases to applications in wound healing and immune-mediated conditions. This innovative approach not only enhances delivery precision but also reduces the need for frequent re-dosing, setting a new standard for cutting-edge dermatological therapies.

While previous reviews have thoroughly addressed hydrogel-based gene delivery in regenerative medicine and the broader scope of polymeric carriers for local nucleic acid delivery (Carballo-Pedrares et al., 2020; Fliervoet et al., 2018), this review distinguishes itself by offering three distinct contributions. First, it focuses exclusively on dermatological applications, providing an in-depth analysis of gene hydrogel platforms for hereditary skin diseases (e.g., epidermolysis bullosa), chronic wounds, and immune-mediated disorders (e.g., atopic dermatitis and psoriasis). Unlike reviews that cover regenerative contexts such as bone or neural tissue repair (Carballo-Pedrares et al., 2020), this review is dedicated to the skin’s unique barriers (e.g., stratum corneum) and disease-specific microenvironments. Second, it integrates both viral and non-viral vector systems within hydrogel platforms, whereas prior studies often focus solely on non-viral approaches (Carballo-Pedrares et al., 2020). Third, it positions gene hydrogels not merely as delivery vehicles but as bioactive platforms that synergize material properties (e.g., three-dimensional extracellular matrix mimicry, stimuli-responsiveness) with gene therapy precision—a dimension less emphasized in general nucleic acid delivery reviews (Fliervoet et al., 2018).

## 2 Overview of hydrogel as gene carrier

### 2.1 Classification and structure of hydrogels

Hydrogels represent a class of three-dimensional (3D) polymeric networks formed by crosslinked hydrophilic macromolecules capable of absorbing substantial amounts of aqueous fluid while resisting dissolution. This unique property arises from their ability to retain water within the interstitial spaces of their porous architecture (Cao et al., 2021). Classification of hydrogels is multifaceted, encompassing criteria such as origin (natural, synthetic, or hybrid), physicochemical properties (e.g., mechanical strength, swelling ratio), ionic characteristics (anionic, cationic, or neutral side groups), crosslinking mechanisms (chemical covalent bonds vs. physical interactions), and responsiveness to stimuli (e.g., pH, temperature, enzymatic activity) (Sun et al., 2021; Chai et al., 2017).

**FIGURE 1:**
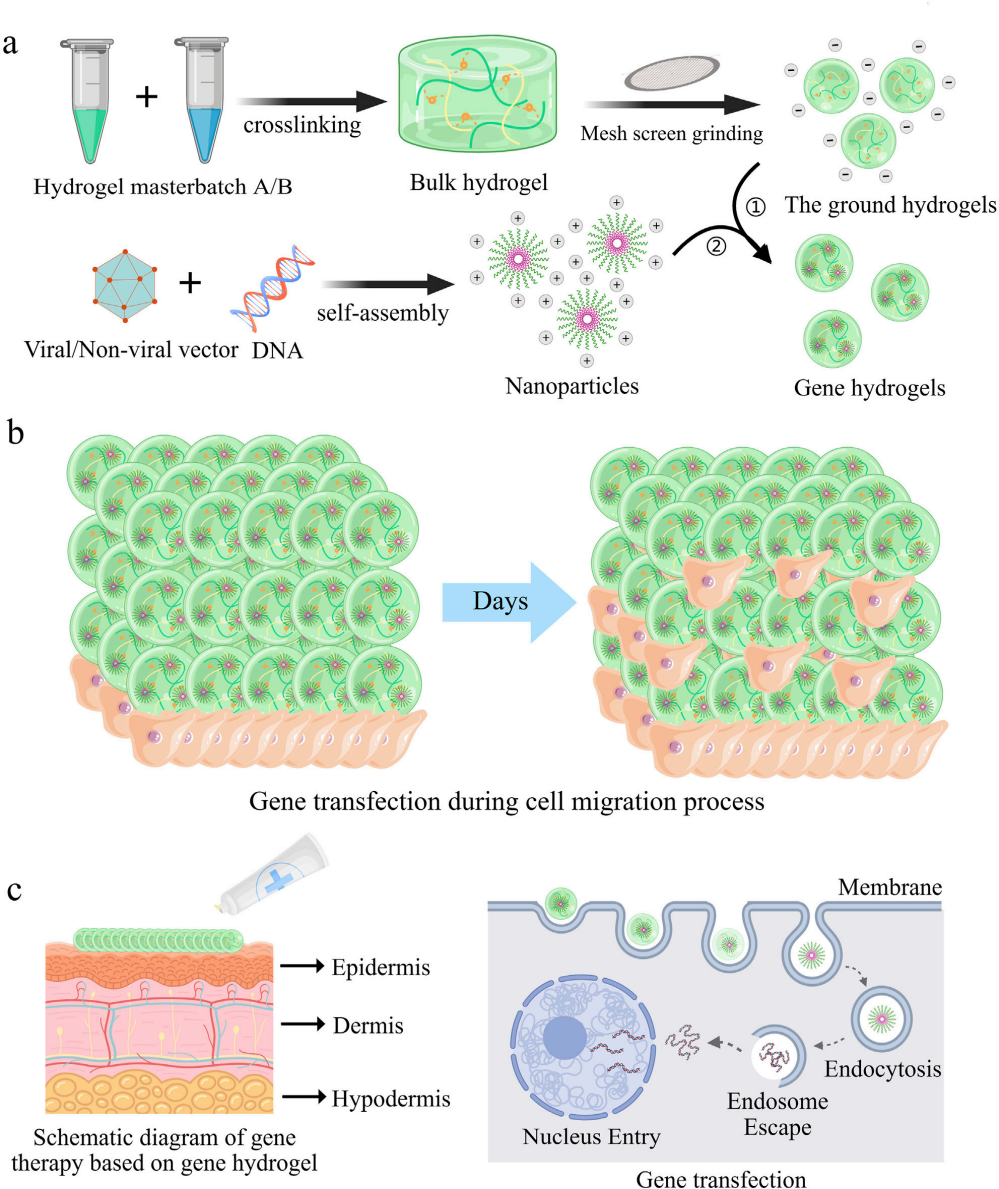
Gene hydrogels mediated gene delivery processes for enhanced gene transfection efficacy and safety. **(a)** Schematic illustration of the preparation of gene hydrogels. **(b)** Schematic representation illustrates cell migration as they enter the interior of the gene hydrogel (Zhang et al., 2024b). **(c)** Schematic illustration of local treatment of skin diseases with gene hydrogels (Zhang et al., 2023).

Hydrogels are synthesized through crosslinking of hydrophilic polymer chains composed of covalently bonded monomeric repeat units. During fabrication, gene-loaded nanoparticles are homogenously dispersed within the hydrosol (pre-crosslinked precursor solution). Subsequent polymerization triggers covalent bond formation between adjacent polymer chains, transforming the hydrosol into a stable gene-embedded hydrogel featuring a three-dimensional entangled network (Figure 1a). This process concurrently creates interconnected micropores (10–500 nm diameter) through controlled interchain spacing, a structural optimization that balances osmotic swelling forces with elastic recoil to recapitulate native extracellular matrix (ECM) biomechanical properties (Mastr et al., 2020; Fang et al., 2020).

In biomedical engineering, hydrogels have emerged as indispensable platforms for drug delivery, regenerative medicine, and wound management. The high porosity inherent to hydrogels facilitates cell infiltration into their interior and enables three-dimensional migration and proliferation. This porous architecture provides the necessary physical framework for cells to interact with receptor-binding ligands presented on the hydrogel surface, establish cell-cell junctions, and enhance adhesion. These combined processes subsequently promote robust cell migration (Figure 1b). ^26^ For subsequent applications of functionalized particles, surface modification is a crucial step, such as the introduction of biofunctional molecules and targeting ligands, to improve their adsorption and endocytosis efficiency in target cells. Gene hydrogels, characterized by shear-thinning and injectable properties, offer versatile delivery options, including use as dressings or injections for localized administration. As demonstrated in Figure 1c, nanoparticles can directly target diseased cells through these hydrogels.

Recent innovations focus on “smart” hydrogels engineered to release genetic payloads (e.g., siRNA, mRNA) in response to pathological stimuli such as dysregulated pH or matrix metalloproteinase (MMP) overexpression. (Cao et al., 2021; Mo et al., 2021; Shan and Wu, 2024). These advancements position hydrogels as next-generation vehicles for spatiotemporally controlled gene therapy, with transformative potential in precision medicine. In conclusion, the developed gene hydrogels are user-friendly, easy to prepare, and biodegradable, and their ability to be directly incubated with cells facilitates *in vitro* evaluation.

### 2.2 Preparation methods of gene hydrogel

In recent years, gene hydrogels have garnered significant attention in the field of controlled drug delivery systems due to their excellent biocompatibility, high capacity for therapeutic molecules, and the slow diffusion characteristics of their elastic networks (Hirsch et al., 2020; Mohamed et al., 2020). Currently, several hydrogel manufacturing techniques are available, including mechanical disruption or stirring (Lacroix et al., 2022), batch emulsification (Lacroix et al., 2022; Guerzoni et al., 2017), microfluidic emulsification (Nakajima et al., 2016), air microfluidics (Chen et al., 2021; Cha et al., 2014), precipitation polymerization (Bustamante-T et al., 2022), and electrospraying (de Rutte et al., 2019).

**FIGURE 2:**
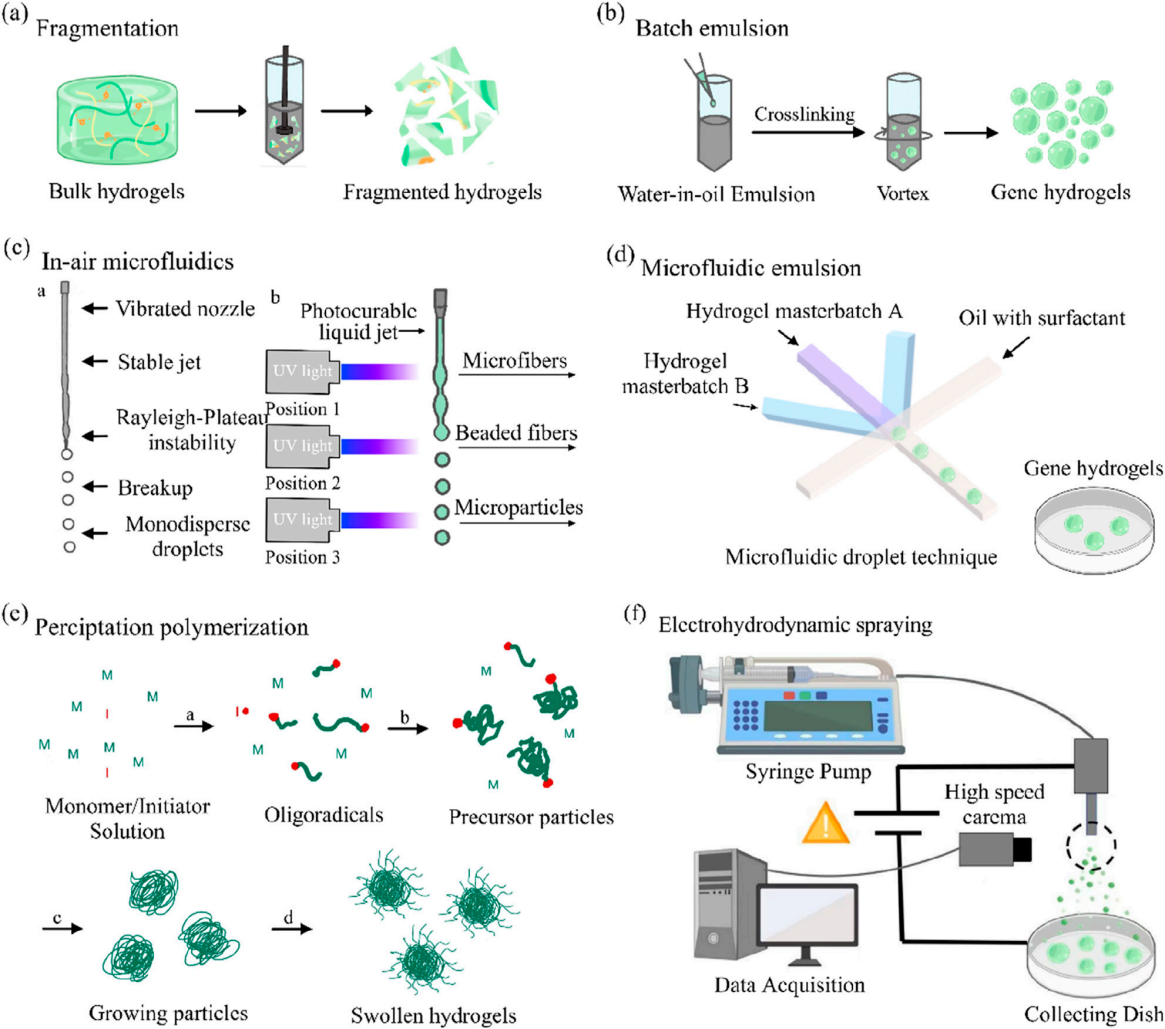
Overview of the different technologies available for gene hydrogels fabrication. **(a)** Gene hydrogels fabrication via fragmentation. **(b)** Gene hydrogels fabrication using a batch emulsion. **(c)** Gene hydrogels fabrication using in-air microfluidics. **(d)** The example of microgel fabrication using a microfluidic emulsion. **(e)** Gene hydrogels fabrication via precipitation polymerization. **(f)** An overview of gene hydrogels fabrication using electrohydrodynamic spraying (Daly, 2023).

Mechanically induced physical fragmentation methods have emerged as a preferred strategy for industrial-scale production due to their operational simplicity and high yield. For instance, extruding pre-crosslinked bulk hydrogels through metallic sieves (Figure 2a) enables rapid production of microparticles with dimensions dictated by the sieve pore geometry. (Lee et al., 2018). Similarly, high-speed shear devices (e.g., homogenizers) can dynamically fragment macroscopic hydrogels into micron-scale particles. (Wolff et al., 2020). However, these techniques exhibit limited capability in controlling particle morphology, often yielding irregularly shaped products, thereby restricting their application in precision drug delivery systems.

Traditional batch emulsification disperses hydrogel precursor solutions into an oil phase under agitation to form droplets (Figure 2b), followed by droplet solidification via photothermal triggering or crosslinker diffusion (Muir et al., 2021). While this method is readily scalable, it suffers from broad droplet size distributions (polydispersity index >0.2), necessitating post-sieving steps to improve uniformity (Widener et al., 2022).

To address oil residue challenges, emerging in-air microfluidics utilizes high-velocity collisions of two liquid streams (containing the precursor and crosslinker) in a gaseous phase (Figure 2c), directly generating monodisperse droplets for *in situ* crosslinking (Kamperman et al., 2018). Recent studies demonstrate that this approach achieves production rates 10–100 times faster than conventional microfluidic chips while eliminating the need for complex washing procedures, offering a cleaner workflow for cell encapsulation applications (Visser et al., 2018).

Microfluidic technology enables precise control of multiphase fluids within microchannels (e.g., flow-focusing or co-flow configurations), allowing the fabrication of gene hydrogels with uniform particle sizes (polydispersity index <0.05) (Figure 2d). (Chen et al., 2024) For instance, linear modulation of particle diameters within the 50–500 μm range can be achieved by adjusting the oil-to-aqueous phase flow rate ratios or capillary dimensions. Notably, the viscosity of the precursor solution must be compatible with the microfluidic chip’s channel dimensions to prevent channel clogging (Mohamed et al., 2020; Chen et al., 2021). Furthermore, parallelized chip designs have successfully enhanced production rates to the gram-per-hour scale, significantly advancing their potential for clinical translation (de Rutte et al., 2019).

Another limitation of emulsions is the presence of oil, which can be challenging to fully remove from final products. To overcome this, oil-free all aqueous two-phase systems can also be employed for gene hydrogels fabrication (Wang et al., 2023).A common method is to form gene hydrogel by precipitation polymerization.

This method involves dissolving monomers, crosslinkers, and initiators in a suitable solvent, followed by polymerization initiated by thermal activation or UV irradiation (Nakano et al., 2020). As polymer chains undergo self-assembly and crosslinking, colloidal particles nucleate and grow until reaching a critical size, after which they precipitate (Figure 2e). (Chen et al., 2024) Although particle size can be modulated by adjusting solvent polarity or monomer concentration, the harsh chemical environment of the reaction system (e.g., free radicals, elevated temperatures) limits its applicability in encapsulating sensitive biomolecules (Hirsch et al., 2020; Jiang et al., 2021).

Electrospraying employs a high-voltage electric field to overcome droplet surface tension, atomizing the precursor solution into charged microdroplets (Figure 2f), which are collected in a crosslinker-containing bath for instantaneous solidification (Xin et al., 2019). For example, sodium alginate solutions can be electrosprayed into a CaCl_2_ bath to form cell-encapsulating microgels (Correia et al., 2019). A key challenge lies in the specialized equipment requirements and the need to optimize the interplay between electric field strength and solution conductivity (Gansa et al., 2018).

It is evident that different preparation methods influence the structure and function of gene hydrogel particles, and the selection of an appropriate manufacturing process can enhance the efficacy of hydrogels in gene delivery. It should be noted that each of the manufacturing methods discussed has been used to produce microgels with sufficient sterility for cell culture and bioprinting applications, and they have shown good potential for gene delivery in dermatological applications (Qazi et al., 2022; Mendes et al., 2021).

## 3 Treatment of hereditary skin diseases with gene hydrogels

### 3.1 Overview of hereditary skin diseases

Hereditary skin diseases are a type of genetic disorder caused by genetic mutations, mainly manifested as abnormalities in skin structure and function. This type of disease usually has genetic susceptibility and is more common in families. Common hereditary skin diseases include but are not limited to epidermolysis bullosa (EB), hereditary vitiligo, keratosis, congenital ichthyosis, and hereditary hemangioma (Yu et al., 2022). Among these, EB serves as a representative condition for understanding the challenges and opportunities in genetic dermatological therapies.

Epidermolysis bullosa (EB) is a heterogeneous group of inherited blistering disorders characterized by skin fragility (Zeng et al., 2021; Zeng et al., 2019; Has et al., 2018; Rashidghamat and McGrath, 2017). Since its initial clinical classification in 1962, EB has been subdivided into four main types along with numerous rare and less-common subtypes (Rashidghamat and McGrath, 2017). Among these, recessive dystrophic epidermolysis bullosa (RDEB) stands as a representative monogenic inherited skin fragility disorder within the EB family. Genetically, RDEB is induced by biallelic single-gene loss-of-function mutations in the *COL7A1* gene, which encodes the skin structural protein type VII collagen (C7). It is well-established that both human keratinocytes and dermal fibroblasts are capable of secreting C7. C7 serves as the principal component of anchoring fibrils (AFs) and furnishes the primary structural connection between the basal membrane zone (BMZ) and the papillary dermis layer of the skin, playing a crucial connecting role at the dermal-epidermal junction. The involvement of this structural protein can give rise to the formation of cracks or blisters beneath the dense plate of the BMZ.

In addition to chronic and recurrent wounds, tissue fibrosis, severe pain, and frequent growth impairments, RDEB patients are confronted with an extremely high risk of developing invasive squamous cell carcinoma. This is attributed to chronic remodeling and enhanced cell proliferation at the lesion site (Castelo et al., 2019). At present, the cure of RDEB still faces significant challenges. Although significant progress has been made in related treatment methods in previous research and practice, such as the use of lentivirus and gamma retroviral vectors to supplement the *COL7A1* gene in keratinocytes and fibroblasts; The use of allogeneic fibroblasts, mesenchymal stromal cells (MSCs), bone marrow transplantation (BMT) (Riedl et al., 2022) and other methods has not yet achieved the ideal effect of completely curing RDEB. This underscores the urgent need for innovative therapeutic strategies, such as localized gene delivery via hydrogels, to address the structural and functional defects caused by *COL7A1* mutations.

### 3.2 Application of gene hydrogels in hereditary skin diseases

Gene hydrogels represent a groundbreaking solution for overcoming the challenges of genetic therapies in hereditary skin diseases. These hydrogels enable localized delivery of genetic material to compromised skin, leveraging the altered skin barrier observed in many genetic dermatoses (Hou et al., 2023; Chen et al., 2023; El Yacoubi and Chbicheb, 2023). Abnormalities in genes encoding epidermal cell components, extracellular lipid matrices, or cell-cell/cell-matrix interactions can increase skin permeability, facilitating the transport of larger molecules, such as gene editors, without requiring external barrier breach (Popp et al., 2024; Tartaglia et al., 2021).

One of the key obstacles to RDEB gene therapy is the large size of the *COL7A1* gene, which encodes type VII collagen and spans 8833 nucleotides (Yu et al., 2022). This size poses significant challenges for efficient gene delivery, particularly through traditional viral vectors, due to payload limitations and reduced transduction efficiency. Despite these challenges, preclinical studies have explored innovative approaches, such as direct intradermal injection of lentiviral vectors expressing C7 or topical delivery of recombinant C7 protein (Castelo et al., 2019; Riedl et al., 2022).

**FIGURE 3:**
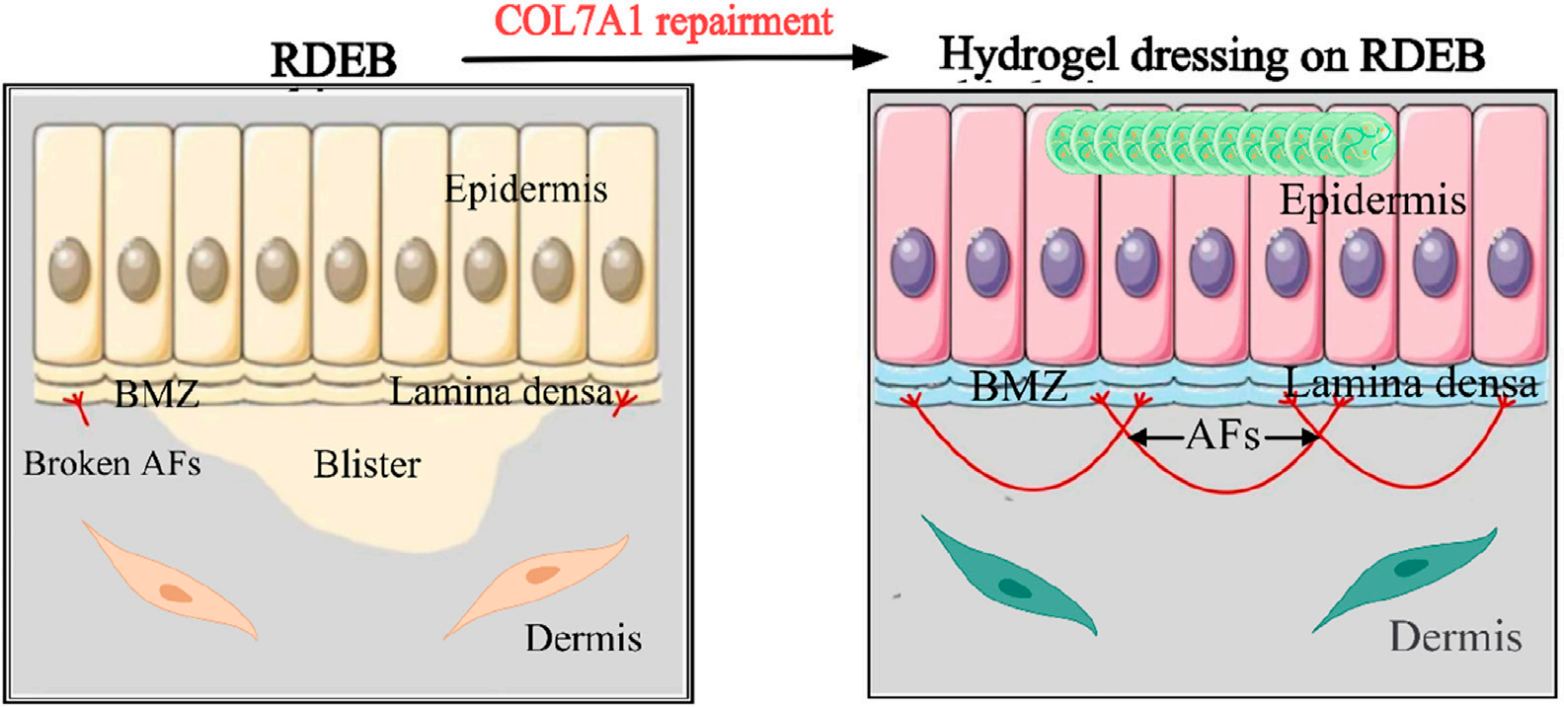
Prospects for the application of gene hydrogel in the field of dermatology. RDEB is caused by *COL7A1* mutations that lead to blistering beneath the lamina densa in BMZ. Keratinocytes and fibroblasts are main sources to secrete C7 which aggregates into AFs (Zeng et al., 2021).

The inherent biocompatibility and minimally invasive nature of hydrogels make them an ideal platform for direct gene therapy application in open wounds of patients with recessive dystrophic epidermolysis bullosa (RDEB). By circumventing the need to breach the intact epidermal barrier, hydrogel-mediated delivery enables localized transfection of dermal cells, stimulating fibroblast-derived type VII collagen (C7) secretion and subsequent anchoring fibril (AF) regeneration to facilitate dermo-epidermal reattachment (Figure 3). A notable advancement in this field is Vyjuvek™ (bercolagene telserpavec), the first FDA-approved *in vivo* localized gene therapy for hereditary skin disorders (Guide et al., 2022). This breakthrough formulation employs a low-immunogenicity herpes simplex virus type 1 (HSV-1) vector (KB103) encoding *COL7A1*, which is admixed with a hydrogel excipient and topically applied to DEB lesions. Clinical validation through Phase I/II trials (NCT03536143, NCT04491604) demonstrated sustained C7 restoration while minimizing systemic vector dissemination through controlled release kinetics (Guide et al., 2022; Gurevich et al., 2022).

Beyond viral vectors, hydrogel systems have been successfully adapted for non-viral gene delivery. Zhang et al. achieved efficient GFP transfection in Hela cells using polyethyleneimine (PEI)-DNA complexes encapsulated within thermoresponsive hydrogels (Zhang et al., 2024a). Similarly, agarose hydrogel-mediated plasmid delivery extended transgene expression duration by 3-fold compared to aqueous injections through enhanced local retention and reduced nuclease degradation (Wu et al., 2023). The utilization of hydrogels as a delivery medium for gene therapy represents a significant advancement over traditional methods such as DNA injection in solution. Hydrogels not only enhance the retention of plasmid DNA at the injection site, thereby prolonging gene expression, but also offer several additional benefits, including improved patient convenience and a reduced risk of injection-related infections (Chamorro et al., 2013). Moreover, gene hydrogels exhibit numerous advantages when compared with other gene therapy products. These benefits include the avoidance of first-pass effects, prevention of wound dressing adhesion, provision of a moist and protective microenvironment that is conducive to wound healing, and alleviation of adverse reactions such as gastrointestinal discomfort (Gurevich et al., 2022).

Following successful application in recessive dystrophic epidermolysis (RDEB), the therapeutic efficacy of gene hydrogels in the treatment of hereditary dermatoses has been thoroughly validated. Their capacity to efficiently and safely deliver the *COL7A1* gene has significantly enhanced skin integrity while minimizing adverse effects. Despite these notable advancements, gene hydrogels still encounter several challenges that require attention for clinical translation. Efforts to optimize delivery systems should prioritize the incorporation of protective agents to enhance nucleic acid stability. Comprehensive evaluations of the off-target effects of gene editing tools and the potential chronic immune responses induced by hydrogel implantation are essential to ensure safety and efficacy. Additionally, the development of precision targeting mechanisms and customizable gene expression profiles tailored to diverse therapeutic needs remains a critical area of investigation. Nonetheless, gene hydrogels present a promising platform for gene delivery, owing to their biocompatibility and sustained-release properties. In the following section, we will investigate the potential application of gene hydrogels in skin wound healing, highlighting their broader therapeutic potential in dermatological treatments.

## 4 Treatment of wound healing with gene hydrogels

### 4.1 The physiological of wound healing

Skin wound healing is a dynamic, multi-phase process that restores tissue integrity through coordinated cellular and molecular interactions (Peña and Martin, 2024). Acute wound healing typically progresses through four overlapping stages: hemostasis, inflammation, proliferation, and remodeling (Sorg and Sorg, 2023). During hemostasis, platelets aggregate at the injury site, forming a fibrin clot that acts as a provisional matrix while releasing growth factors (e.g., PDGF, TGF-β) to recruit immune cells (Freedman et al., 2023). The subsequent immune phase (24–72 h post-injury) involves neutrophils clearing debris and macrophages polarizing from pro-immune (M1) to anti-immune (M2) phenotypes, resolving inflammation and initiating tissue repair (Novak and Koh, 2013). In the proliferative phase (3–21 days), keratinocytes migrate across the wound bed via integrin-mediated interactions with the extracellular matrix (ECM) (Santoro and Gaudino, 2005), while fibroblasts synthesize collagen-rich granulation tissue under the regulation of TGF-β and VEGF (Yao et al., 2024), Finally, remodeling (weeks to years) ensures ECM maturation through collagen crosslinking and realignment mediated by matrix metalloproteinases (MMPs) and tissue inhibitors of metalloproteinases (TIMPs) (Lin et al., 2023).

In contrast, chronic wounds (e.g., diabetic ulcers, venous leg ulcers) fail to progress through these stages due to persistent inflammation, hypoxia, or microbial biofilms (Talbott et al., 2022). Prolonged M1 macrophage dominance perpetuates oxidative stress and excessive protease activity (e.g., MMP-9), degrading ECM components and growth factors (Mazurek et al., 2022), Chronic hypoxia, often linked to microvascular dysfunction in diabetes, impairs fibroblast proliferation and angiogenesis. Additionally, senescent fibroblasts in aged or diabetic skin exhibit reduced responsiveness to growth signals, further delaying re-epithelialization (Demaria et al., 2017). Bacterial biofilms, particularly *Staphylococcus aureus* and *Pseudomonas aeruginosa*, exacerbate inflammation and resist immune clearance through quorum sensing (Sharifiaghdam et al., 2022). In summary, skin wound healing is a highly organized physiological process that involves the synergistic effects of multiple cell types and molecular mechanisms. A deeper understanding of this process can help develop more effective wound treatment strategies.

### 4.2 Application of gene hydrogels in wound treatment

Gene hydrogels represent a transformative approach to enhancing skin wound healing by integrating the regenerative properties of hydrogels with targeted gene delivery systems (Zhao et al., 2017; Gong et al., 2013; Guo et al., 2013). Specifically, the hydrogel matrix provides a porous structure and an appropriate swelling ratio, which allows for the presence of oxygen, absorption of exudates, and maintenance of a moist healing environment (Zhu et al., 2025), thereby promoting wound healing (Elhabal et al., 2023). Additionally, hydrogel adhesives can isolate external bacterial clones, promote gas exchange, and inhibit the proliferation of anaerobic bacteria (Yampolsky et al., 2024). Unlike traditional wound dressings (e.g., gauze and cotton wool), hydrogel dressings loaded with bioactive molecules exhibit ideal biological activity by releasing encapsulated drugs from the hydrogel matrix (Gopinath et al., 2004).

**FIGURE 4:**
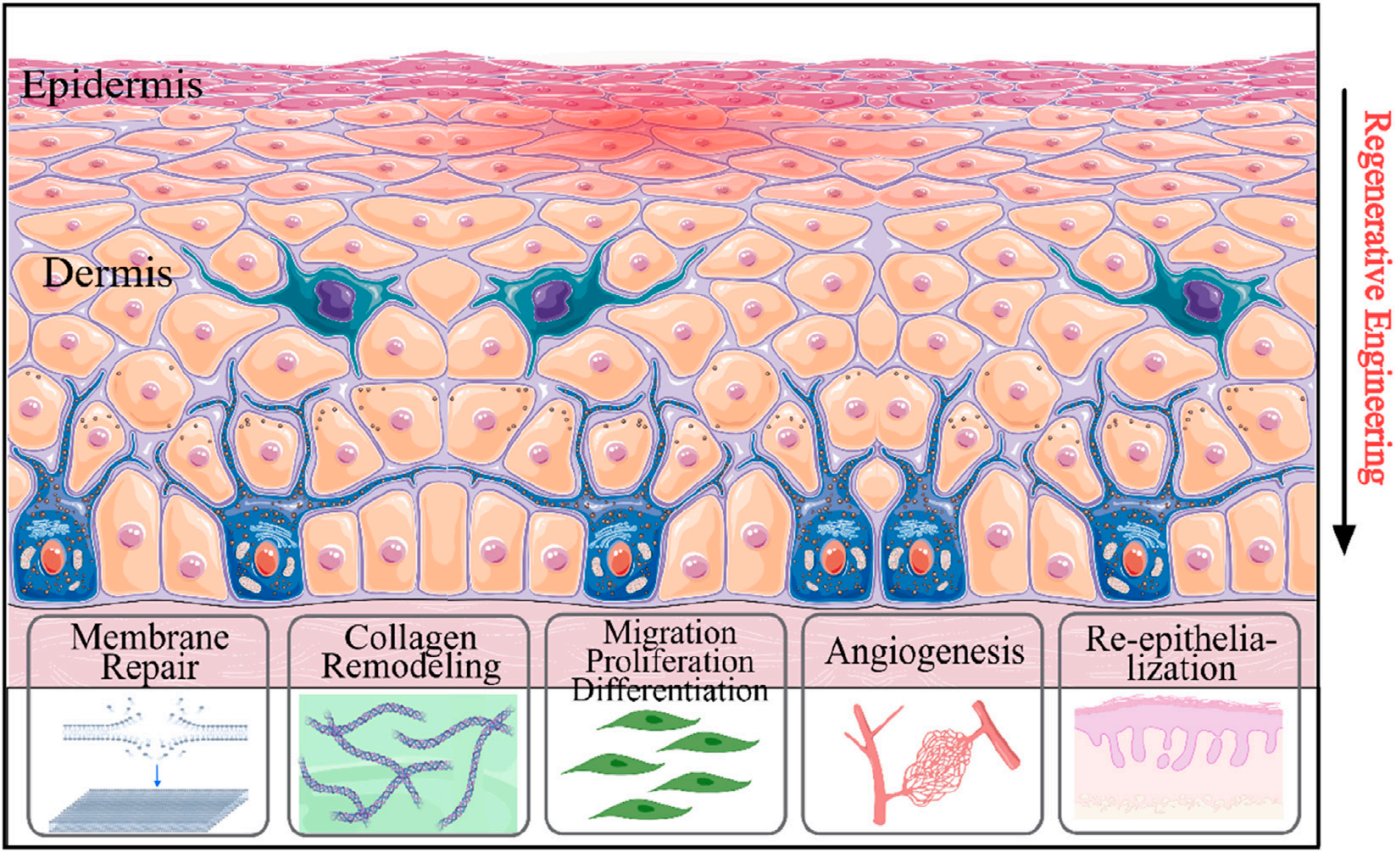
Schematic illustration of the involvement of gene therapy approaches in wound healing process. Gene therapy systems enhanced re-epithelialization, cell attachment, genes linked to angiogenesis, collagen remodeling, cell differentiation, and cell migration.

Compared with traditional hydrogel dressings that primarily offer moisturizing and physical protection (Peng et al., 2022), gene hydrogels address potential molecular pathological issues through local nucleic acid delivery. These hydrogels can be modified to release plasmid DNA, siRNA, or miRNA that regulate critical healing pathways. These gene-modified hydrogels have been demonstrated to significantly mitigate inflammatory responses during wound healing, minimize drug-induced cytotoxicity to host cells, and expedite tissue regeneration (Figure 4). (Elhabal et al., 2023) For instance, the hydrogel loaded with plasmid DNA encoding VEGF significantly enhanced angiogenesis by 2.5-fold by sustaining the expression of growth factors from 7 days (free vector) to 21 days (Lou et al., 2020). Similarly, chitosan hydrogels loaded with miR-29b accelerated wound closure in diabetic mice by 40% through collagen regulation (Kim et al., 2022).

The latest innovative hydrogels employ stimulus-responsive polymers that react to changes in pH or enzyme activity at the wound site to release therapeutic genes (Alkekhia et al., 2022). Biocompatibility studies have demonstrated that even at high nucleic acid loading concentrations, the cytotoxicity of human skin fibroblasts (HDFs) is minimal, supporting the potential for clinical translation (Lee et al., 2021). However, optimizing transfection efficiency without viral vectors remains a challenge. For example, a positively charged hydrogel matrix modified with polyethyleneimine (PEI) achieved 65% siRNA uptake in HDFs by enhancing intracellular escape (Fattahi et al., 2024).

Despite the promising preclinical results, scaling up presents challenges, including sterilization stability and cost-effective manufacturing (Carballo-Pedrares et al., 2020). The imbalance between the mechanical strength and degradation rate of existing hydrogels may affect the long-term efficacy of wound healing. Future research should focus on personalized hydrogel platforms to adapt gene delivery profiles to individual wound microbiomes and healing biomarkers. With continuous development, gene-activated hydrogels have the potential to revolutionize chronic wound management by providing precise treatment that meets the requirements of molecular and structural healing (Carballo-Pedrares et al., 2020).

## 5 Treatment of immune related skin diseases with gene hydrogels

### 5.1 Immune related skin diseases: mechanisms and challenges

Immune related skin diseases constitute a growing global health crisis, affecting approximately 20%–30% of the world’s population (Hay et al., 2014). These conditions, characterized by dysregulated interactions between innate and adaptive immunity, impose profound physical, psychological, and economic burdens. Among these diseases, atopic dermatitis (AD) and psoriasis (PsO) stand out as archetypal disorders with distinct immunological mechanisms, yet overlapping societal impacts.

Atopic dermatitis (AD) is the most common chronic pruritic immune skin disease (Langan et al., 2020), characterized by inflammation, impaired skin barrier function, and ecological imbalance, leading to the formation of itchy and eczema areas (Czarnowicki et al., 2019). Its pathogenesis depends on the Th2/Th22 polarized immune axis, which damages the skin barrier and maintains itching, exacerbating skin barrier dysfunction and promoting ecological imbalance. Targeting these pathways by blocking IL-4 (Renert-Yuval and Guttman-Yassky, 2020), IL-13 (Renert-Yuval and Guttman-Yassky, 2020), IL-31 (Thyssen and Schmid-Grendelmeier, 2023) and inhibiting Janus kinase activity (Thyssen and Thomsen) has been shown to effectively improve the prognosis of AD patients. Clinically, AD presents as a vicious pruritus-scratch cycle, with lichenification and excoriations predominantly occurring in skin folds (e.g., axillae, neck) – regions subject to complex and extensive mechanical deformation (Bieber, 2022). Consequently, hydrogels designed for this application must exhibit exceptional softness combined with high tensile strength and toughness to withstand these stresses. Secondly, unconscious scratching in AD patients poses a risk of localized damage to the dressing, necessitating intrinsic self-healing capabilities in the hydrogel material. Finally, robust tissue adhesion is essential to ensure stable, long-term adherence to the dynamic skin surface, eliminating the need for supplementary fixation methods such as medical tapes or gauze.

**FIGURE 5:**
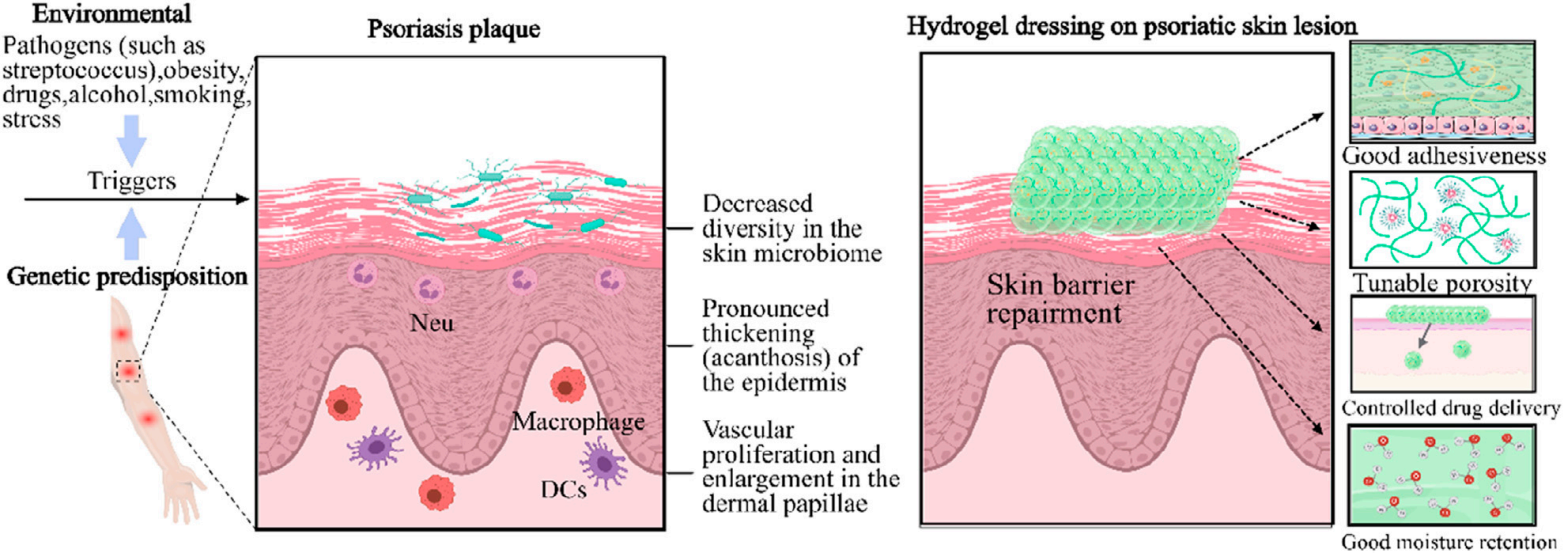
Distinctive therapy of gene hydrogels as a topical therapeutic platform for inflammatory skin diseases. The development of a psoriasis plaque involves the participation of plasmacytoid dendritic cells and type I interferons, which lead to a marked thickening of the epidermis. DC: dendritic cell. Neu: neutrophil. Gene hydrogels feature tunable porosity, excellent adhesiveness, controlled drug delivery ability, and moisturizing capability (Cao et al., 2024).

Unlike AD, psoriasis (PsO) is a systemic IL-17/IL-23 driven disease with a strong genetic component (HLA-C*06:02 confers 40%–50% heritability) (Nakats et al., 2016). Pathologically speaking, IL-23 derived from dendritic cells activates Th17 cells to excessively produce IL-17A and IL-22, leading to excessive proliferation of keratinocytes through STAT3 and NF - κ B pathways, resulting in typical psoriasis plaques: well-defined mica scale erythema lesions (Figure 5). (Hawkes et al., 2017) In addition to skin involvement, 30% of psoriasis patients also develop psoriatic arthritis (PsA) (Ogdie et al., 2015). Moreover, psoriasis patients are more than three times more likely to suffer from depression and anxiety than normal individuals, mainly due to the itching, pain, and social stigma associated with chronic plaques or psoriasis vulgaris caused by a combination of genetic susceptibility and environmental factors such as streptococcal infection, stress, smoking, obesity, and alcohol consumption (Michalek et al., 2017).

Both AD and PsO highlight the importance of personalized treatment approaches. JAK inhibitors (e.g., upadatinib for AD) and IL-23p19 antagonists (e.g., risankizumab for PsO) have exhibited potential in clinical trials for the targeted regulation of immune pathways (Navarro-Triviño et al., 2023). Nevertheless, conventional treatment methods, such as drug therapy (including topical and oral medications) and physical therapy, while achieving certain therapeutic effects, are also associated with several limitations. These include adverse effects like skin atrophy, pigmentation, and an elevated risk of skin cancer (Rahmatpour et al., 2023). To overcome these challenges, gene hydrogel emerges as a promising novel strategy for treating immune related skin diseases. It offers several benefits in the treatment of immune related skin diseases, including precise targeting, sustained drug release, excellent biocompatibility, promotion of tissue repair, strong controllability, and convenient local administration (Grän et al., 2020; Jin et al., 2023; Zouboulis et al., 2022).

### 5.2 Research findings of gene hydrogels in immune related skin diseases

Recent progress has positioned hydrogels as a revolutionary platform for the treatment of chronic inflammatory skin diseases such as atopic dermatitis (AD) and psoriasis. The inherent moisturizing ability of hydrogel is used to solve the key AD pathological problem of xerosis by maintaining 85%–92% skin water within 24 h (Ha et al., 2022). Hydrogels also allow precise control of drug release rate and duration. For example, MTX-NPs loaded hydrogel showed 73% ± 1.21% continuous drug release within 48 h, but for the treatment of AD/PsO, the release rate and total amount may need to be adjusted according to the condition (Asad et al., 2021).

AD, characterized by Th2-driven inflammation and skin barrier dysfunction, necessitates hydrogels that simultaneously modulate immunity and restore hydration. Some studies have shown that the gene hydrogel containing anti RelA siRNA and functional peptide has therapeutic effect in the model of atopic dermatitis (AD) in mice (Kanazawa et al., 2015). After local application of hydrogel containing functional peptide, siRNA is more widely delivered to the application site of AD induced mouse ear skin than the preparation without functional peptide, and can improve the ear thickness and clinical skin severity of AD induced mice. Another adhesive composite hydrogel patch is composed of poly (N-2,3-dihydroxypropyl asparagine) (PDHPA), polyasparagine derivatives and mesoporous silica nanoparticles (MSNs), because MSNs can improve the mechanical properties, adhesion properties and self-healing rate of hydrogels, and can load hydrophobic drugs such as dexamethasone, successfully reducing the severity of atopic dermatitis in the mouse model (Kim et al., 2023). Furthermore, leveraging the observed vicious pruritus-scratch cycle in AD, Jia et al. (2023) developed an innovative boronate-based hydrogel dressing exhibiting adhesion, stretchability, and self-healing properties. This hydrogel simultaneously encapsulated polydopamine nanoparticles (PDA NPs) for reactive oxygen species (ROS) scavenging and liposome-embedded hydrophobic focal adhesion kinase inhibitor (FAKi-lipo) for FAK inhibition, constituting a synergistic therapeutic strategy against AD. During the experimental phase, mice were randomized into treatment groups and subjected to different interventions. A sustained allergen challenge and scratching behavior were recapitulated through continuous application of 25  μM MC 903 combined with mechanical stimulation. Following a 10-day treatment regimen, immunohistochemical analysis revealed a striking reduction in pFAK expression within the skin tissue of the hydrogel-treated group. This finding demonstrates the effective modulation of FAK phosphorylation in the skin achieved via FAKi-lipo delivery mediated by the hydrogel. Moreover, dermatitis severity scores indicated significant clinical improvement in the hydrogel-treated group (3.14 ± 0.34) compared to the ADscratch control group (9.29 ± 0.29). This amelioration is likely attributable to the combined effects of ROS scavenging by the hydrogel components and FAK inhibition, collectively mitigating acute inflammation.

While AD is dominated by Th2-mediated inflammation, psoriasis presents a distinct immunological landscape driven by IL-17/IL-23 axis. This paradigm shift has inspired hydrogel designs targeting keratinocyte hyperproliferation and pro-inflammatory pathways. In this context, the hydrogel containing ZnO/Ag nanoparticles and methotrexate (MTX) showed dual anti proliferation and antioxidant effects, realizing the combined multi-target treatment of psoriasis. ZnO/Ag nanoparticles can reduce the innate cytokine profile by inactivating p65 in pro-inflammatory macrophages, and eliminate the secretion of adaptive cytokines in keratinocytes (KCs) by downregulating ROS mediated STAT3 cyclin D1 signaling, thereby exhibiting self therapeutic effects. Local application of the hydrogel on the mouse model of imiquimod (IMQ) induced psoriasis can achieve better anti psoriasis effect (Xu et al., 2022). Beyond synthetic nanoparticles, natural product-based hydrogels have also demonstrated efficacy. For instance, curcumin loaded hydrogel was applied to the model of psoriasis induced by IMQ in mice. At 12 weeks, PCR showed that compared with the normal mouse group, the mRNA levels of IL-1 β, IL-6, IL-17F, IL-22, and IL-23 in the skin of the IMQ alone group were significantly increased. Compared with IMQ alone group, the mRNA levels of these six cytokines in curcumin gel group and tacrolimus group were significantly reduced (p < 0.05) (Sun et al., 2017). This has guiding significance for the treatment of diseases.

Moreover,a recent advance in hydrogel-based psoriasis therapy is the SJMHE1-loaded hydrogel developed by Liu et al. (2025) SJMHE1, a 24-amino-acid peptide derived from Schistosoma japonicum egg and adult worm antigens, induces CD4^+^CD25^+^ regulatory T (Treg) cells and M2 macrophages in a TLR2-dependent manner, thereby suppressing delayed-type hypersensitivity (DTH). In their experimental design, Liu et al. established an IMQ-induced murine psoriasis model and randomized the mice into treatment groups. The vehicle control group received no treatment, while the SJMHE1-gel group received a daily topical dose of 20 μg SJMHE1 for 14 days. Histopathological evaluation (H&E staining) post-treatment revealed significant improvement in the SJMHE1-gel group, characterized by reduced epidermal thickness, diminished parakeratosis and hyperkeratosis, improved acanthosis, and decreased dermal inflammatory cell infiltration. Furthermore, SJMHE1 treatment markedly reduced the number of Ki67-positive cells, indicating inhibition of aberrant keratinocyte proliferation and differentiation. Immunohistochemical analysis demonstrated that SJMHE1-gel therapy significantly downregulated IMQ-induced IL-17 mRNA expression. Concurrently, Western blot analysis revealed elevated expression of p-p65 and p-STAT3 in the control group, which was substantially attenuated following SJMHE1 hydrogel treatment (Lim et al., 2024). Collectively, these findings suggest that SJMHE1 ameliorates psoriatic skin lesions by inhibiting the activation of the NF-κB and STAT3 signaling pathways, consequently suppressing pro-inflammatory cytokine secretion.

Although hydrogels may become a new generation of drugs for the treatment of AD/PsO, there are still some problems to be solved (Sun et al., 2017). The genetic heterogeneity between patients requires carriers to have dynamic regulatory abilities, but current technology has not yet achieved precise adaptation (Algahtani et al., 2020). Materials with better biocompatibility and durability, such as silk fibroin silk fibroin (SC), should be selected while ensuring treatment effectiveness to reduce skin irritation and adverse reactions. Design hydrogels that can accurately control drug release according to disease status or external stimuli (such as temperature, pH value, etc.). Combine nanotechnology, such as nanoparticles, nano lotion, etc., to improve the skin permeability of drugs. For example, curcumin is made into nano lotion and converted into nano latex gel, which increases the penetration of curcumin. The most important thing is that for AD/PsO patients with different severity, the drug type, dosage and immune regulatory components in the hydrogel should be properly adjusted to achieve personalized treatment.

Building upon the challenges outlined for AD/PsO therapy—including genetic heterogeneity, material biocompatibility, and personalized dosage optimization—Table 2 consolidates key gene hydrogel systems across dermatological applications. This synthesis distills material compositions, genetic payloads, and preclinical outcomes, while highlighting shared translational bottlenecks discussed throughout Sections 3–5. By cross-referencing strategies for hereditary disorders, chronic wounds, and immune-mediated diseases, the table not only encapsulates technological innovations but also foregrounds unmet needs in stimulus-responsive design and scalable manufacturing.

**TABLE 2:** Comparative overview of gene hydrogel systems for dermatological applications.

Application domain	Gel/Polymer type	Preparation method	Genetic material	Target skin condition	Key outcomes/Findings	Limitations/Challenges	Ref.
Hereditary Disorders	HSV-1 vector + hydrogel excipient	Mixing with hydrogel excipient	COL7A1 plasmid (via HSV-1)	Recessive dystrophic EB (RDEB)	Sustained type VII collagen restoration; first FDA-approved *in vivo* gene therapy for EB	Potential host immune response to HSV-1 vector; limited transgene cargo capacity	Guide et al. (2022)
	PEI-DNA + thermoresponsive hydrogel	Crosslinking with temperature	GFP plasmid	*In vitro* cell transfection	Efficient transfection in Hela cells; prolonged transgene expression vs. aqueous solutions	PEI’s potential cytotoxicity at high concentrations; variable transfection efficiency	Zhang et al. (2024b)
	Agarose hydrogel	Precipitation polymerization	COL7A1 mRNA	Patient-derived keratinocytes	3-fold extended expression duration; reduced nuclease degradation	Limited mechanical strength; potential for burst release at initial stages	Wu et al. (2023)
Chronic Wounds	VEGF-loaded hydrogel	Microfluidic emulsification	VEGF plasmid	Diabetic ulcers	2.5-fold enhanced angiogenesis; sustained growth factor release (21 days vs. 7 days)	Non-viral vector’s transfection efficiency lower than viral systems; potential off-target angiogenesis	Lou et al. (2020)
	Chitosan hydrogel	Batch emulsification	miR-29b	Diabetic mouse wounds	40% accelerated wound closure via collagen regulation	pH-dependent degradation rate; potential immunostimulation in chronic inflammation	Kim et al. (2022)
	PEI-modified cationic hydrogel	Electrospraying	siRNA	Human skin fibroblasts (*in vitro*)	65% siRNA uptake efficiency; improved intracellular escape	Electrospraying equipment complexity; potential aggregation of cationic polymers *in vivo*	Fattahi et al. (2024)
Immune-Related Diseases	Anti-RelA siRNA + functional peptide hydrogel	Mechanical disruption	Anti-RelA siRNA	Atopic dermatitis (AD)	Reduced ear thickness and clinical severity in AD mouse model; enhanced siRNA delivery	Heterogeneous particle size from mechanical fragmentation; limited long-term stability	Kanazawa et al. (2015)
	PDHPA-MSNs composite hydrogel	In-air microfluidics	Dexamethasone (loaded in MSNs)	AD mouse model	Improved mechanical properties; reduced AD severity via ROS scavenging and FAK inhibition	MSN synthesis complexity; potential accumulation of inorganic nanoparticles in tissue	Kim et al. (2023)

## 6 Summary

### 6.1 Conclusion

This review uniquely advances the field by presenting the first comprehensive synthesis of gene hydrogel applications in three key dermatological domains: hereditary disorders, chronic wounds, and immune-related diseases. Unlike prior reviews focusing on regenerative medicine or generic nucleic acid delivery, we highlight how gene hydrogels tackle skin-specific barriers—from stratum corneum penetration to inflammation modulation. By linking disease molecular pathologies to tailored hydrogel strategies (e.g., pH-responsive release for psoriasis), this work delivers unparalleled disease-focused depth.

Gene hydrogels, as an emerging class of gene delivery materials, integrate the precision of gene therapy with the functional versatility of hydrogels, offering transformative potential for treating hereditary skin diseases, chronic wounds, and immune-mediated dermatoses. However, their clinical translation faces critical challenges: inherent batch-to-batch variability in hydrogel synthesis compromises manufacturing consistency and therapeutic reproducibility; synthetic polymer components raise immunogenicity risks, necessitating rigorous biocompatibility evaluations; scalability limitations hinder large-scale production of clinical-grade materials, particularly for personalized therapies; balancing mechanical strength with controlled degradation kinetics poses material performance trade-offs, risking premature breakdown or reduced therapeutic durability; and genetic heterogeneity among patients demands dynamically regulated delivery systems, which current technologies inadequately address. These multifaceted challenges underscore the urgent need for optimization to advance gene hydrogels from bench to bedside.

To address the aforementioned challenges, potential solutions are as follows: (i) Implementing microfluidic-based synthesis platforms (e.g., in-air microfluidics or parallelized chip designs) enables precise control over hydrogel architecture, thereby minimizing batch-to-batch physicochemical variability. (ii) Developing bioinert hydrogel matrices from natural polymers (e.g., silk fibroin, hyaluronic acid) or surface-modifying synthetic polymers with anti-inflammatory moieties (e.g., arginine-glycine-aspartic acid peptides) mitigates immune activation. (iii) Adopting 3D bioprinting or electrohydrodynamic spraying technologies achieves high-throughput fabrication while ensuring structural uniformity for scalable production. (iv) Engineering dynamic covalent hydrogels (e.g., disulfide or imine bond crosslinking). to carry gene payloads allows exploration of tunable degradation kinetics that match tissue regeneration rates. (v) Integrating single-cell sequencing and machine learning to design patient-specific hydrogel formulations—such as core-shell particles co-encapsulating gene editors and small-molecule adjuvants—enables personalized regulation of therapeutic gene expression, addressing interpatient genetic heterogeneity.

### 6.2 Future perspectives

Beyond the aforementioned solutions for gene hydrogels, future research may additionally focus on the following directions: (i) Development of intelligent materials: Designing dynamic covalent hydrogels or light/heat-responsive vectors to enable real-time regulation of gene release. For instance, near-infrared-responsive hydrogels can be combined with optogenetic technology to achieve on-demand activation of therapeutic genes. (ii) Multidisciplinary fusion innovation: Integrating single-cell sequencing and machine learning to identify the optimal combinations of genetic materials. Utilizing 3D bioprinting to construct patient-specific skin models can guide the development of personalized treatment plans. (iii) Upgrade of delivery systems: Developing core-shell structured particle hydrogels to synchronously deliver gene drugs and small molecule adjuvants (e.g., anti-fibrosis drugs), thereby enhancing efficacy through multiple pathways. Gene hydrogels signify a paradigm shift in skin disease treatment from “symptom control” to “cause repair.” With the cross-integration of materials science and gene editing technology, future breakthroughs are anticipated in the intelligence, personalization, and multifunctionality of carriers.
